# Sea buckthorn, its bioactive constituents, and mechanism of action: potential application in female reproduction

**DOI:** 10.3389/fendo.2023.1244300

**Published:** 2023-11-07

**Authors:** Michal Mihal, Shubhadeep Roychoudhury, Alexander V. Sirotkin, Adriana Kolesarova

**Affiliations:** ^1^ Institute of Applied Biology, Faculty of Biotechnology and Food Sciences, Slovak University of Agriculture in Nitra, Nitra, Slovakia; ^2^ Department of Life Science and Bioinformatics, Assam University, Silchar, India; ^3^ Department of Zoology and Anthropology, Faculty of Natural Sciences, Constantine the Philosopher University in Nitra, Nitra, Slovakia; ^4^ AgroBioTech Research Centre, Slovak University of Agriculture in Nitra, Nitra, Slovakia

**Keywords:** *Hippophae rhamnoides*, isorhamnetin, quercetin, reproduction, female, proliferation, apoptosis, cancer

## Abstract

Sea buckthorn (*Hippophae rhamnoides* L.) is a flowering shrub, and its berries have been utilized for decades as a raw ingredient in cuisines and herbal remedies. This evidence-based study focuses on its key bioactive constituents, and mechanism of protective effects with a focus on female reproductive processes. Parts of the plant contain phenols, carotenoids (lycopene, carotene, lutein, and zeaxanthin), flavonoids (isorhamnetin, quercetin, glycosides, and kaempferol), tocopherols, sterols, polyunsaturated fatty acids, minerals, vitamins, omega 3, 6, 9 and rare omega 7 fatty acids etc. Key polyphenolic flavonoids such as isorhamnetin and quercetin are believed to be mainly responsible behind its health benefits (against cardiovascular diseases, metabolic syndrome, obesity etc.) through properties including anti-cancer, antioxidant, and anti-inflammatory activities. These sea buckthorn constituents appear to mediate healthy ovarian cell proliferation, death, and hormone release, as well as decrease ovarian cancer possibly through apoptosis, and hormonal (estrogen) release. Thus, sea buckthorn and its bioactive ingredients may have potential in the management of gynecological problems such as uterine inflammation, endometriosis, and easing symptoms of vulvovaginal atrophy in postmenopausal women (by targeting inflammatory cytokines and vascular endothelial growth factor – VEGF). Apigenin, myricetin, and luteolin have also been recommended as prospective ovarian cancer preventative and adjuvant therapy options as they can inhibit ovarian cancerogenesis by triggering apoptosis and halting the cell cycle in ovarian tumors. Furthermore, its oil (containing carotenoid, sterol, and hypericin) has been speculated as an alternative to estrogen replacement therapy for postmenopausal women particularly to improve vaginal epithelial integrity. However, it is uncertain whether steroid hormone receptors, reactive oxygen species (ROS), and inflammatory regulators are actually behind sea buckhorn’s actions. Sea buckthorn, and its compounds’ health promoting potential warrants further validation not just *in vitro* and in animal research, but also in clinical trials to identify and/or standardize optimal methods of delivery of biologically active molecules.

## 1 Introduction

Nowadays, population diseases are becoming more and more widespread. Many factors are responsible for this phenomenon, be it stress, free radical production or lifestyle. The maintenance of the body’s redox status has been credited in large part to hormones (1). For example, estradiol has been proven to have a greater impact on the oxidant-antioxidant balance in numerous tissues. Although progesterone lacks the typical chemical structure of an antioxidant, it appears to lessen oxidative damage when present at high amounts (2, 3). Despite these findings, it is important to supplement dietary polyphenols and phytonutrients commonly found in plants. This evidence-based study focuses on the bioactive constituents, and mechanism(s) of protective effects of sea buckthorn (*Hippophae rhamnoides* L.) with a focus on female reproductive processes. Sea buckthorn is a flowering shrub belonging to the family *Elaeagnaceae*. It is native to cold temperate regions of Eurasia (4). This economically and ecologically important medicinal plant is a winter hardy, dioecious, wind-pollinated multipurpose shrub bearing yellow or orange berries with nitrogen-fixing ability. It grows widely in cold regions of the Indian Himalayas, China, Russia, and many other North American and European countries. Due to its enormous potential as a bioresource for land restoration, preventing soil erosion, and its variety of uses, it is frequently referred to as “cold desert gold” (5). Because of its usage in pharmaceutical and cosmetic compounds, as a source of energy, soil enhancer, and as rich nutritional content, sea buckthorn has a high economic worth (6). Almost all parts of the plant may be used as food, firewood, traditional medicine, and a fence. This plant includes many chemical compounds with a range of biological and medicinal effects (7). Sea buckthorn has been used for centuries as a medicinal and nutritional supplement across Asia and Europe (8). Its berries have been utilized for decades in many parts of the world as a raw ingredient in cuisines and herbal remedies. Berries’ therapeutic and/or nutritional properties make them an affordable source of raw material for the pharmaceutical industry, which benefits mankind (7). As herbal dietary supplements are used more often in many nations, it is crucial to regulate food items that include these ingredients. However, there is little information on the plant and its extracts’ safety assessment (8). Several medicinal benefits of this plant have been well documented, including antioxidant, antitumor, hepatoprotective, or immunomodulating activities (9, 10). Medicinal plants have widely been acknowledged to be the basis for active principles for both therapeutic and preventive measures. Recently, a range of pharmaceuticals have been reported for their antioxidant and anticancer potentials and regulation of hormonal levels to the advantage of management of several disease conditions. Alkaloids, phenols, and acetogenins isolated from graviola (*Annona muricata*) have not only shown promise as possible cancer-fighting agents but also in modulation of cellular proliferation and necrosis. This plant’s extract has been reported to downregulate anti*-*apoptotic genes involved in the pro-cancer metabolic pathways and decreasing the expression of proteins involved in cell invasion and metastasis while upregulating proapoptotic genes and genes involved in the destruction of cancer cells (11). Plant-derived polyphenols including resveratrol, curcumin, quercetin, green tea flavonoids, caffeic acid phenethyl ester, luteolin, xanthohumol, genistein, alpinetin, proanthocyanidins, anthocyanins, silymarin as well as phenolic substances such as thymol, alkaloids like berberine, storage polysaccharides like tamarind xyloglucan, and antioxidant hormones (e.g. melatonin) have been reported to target cellular signaling pathways to reduce intestinal inflammation occurring with inflammatory bowel disorder (1). Plant-based inhibitors of dipeptidyl peptidase-IV (an enzyme that triggers the catalysis of insulinotropic hormones by abating endogenous insulin levels and elevating glucose levels in blood plasma) such as alkaloids, phenolic acids, flavonoids, quercetin, and coumarin have recently been proposed as anti-diabetic by virtue of their hypoglycemic and antioxidative properties (12).

However, a summary of sea buckthorn’s physiological and therapeutic effects on female reproductive systems and/or diseases is still lacking. The latest research on sea buckthorn’s components, characteristics, physiological effects, and therapeutic uses is reviewed together with their methods of action at multiple regulatory levels, with a focus on female reproductive systems.

The aim of this study was to review the progress in the research on sea buckthorn [regardless of the Latin name, *Hippophae rhamnoides* (including all subspecies)] and its potential application in female reproduction made from 2015 to 2023. Publications about the biological activity of sea buckthorn extracts and their constituents and the mechanism(s) of action have also been described. However, the number of available articles on pharmacological properties of different extracts or natural products from this plant is very large, hence the concerned section of the article only highlights some important aspects of research made during the last five years. The literature search was performed using Google Scholar, PubMed, and Scopus search engines, with a time limit from 2015 to 2023. Keywords “sea buckthorn” or ‘rhamnoides’ were combined with ‘flavonoids’, ‘isorhamnetin’, “phenolic compounds”, ‘quercetin’, ‘ovarian tumor’, ‘female reproduction’, “anti-inflammatory activity”, “anticancer activity”, “antiviral activity” etc. Finally, 84 original articles from this period were included in this review.

## 2 Major bioactive constituents

**Figure 1:**
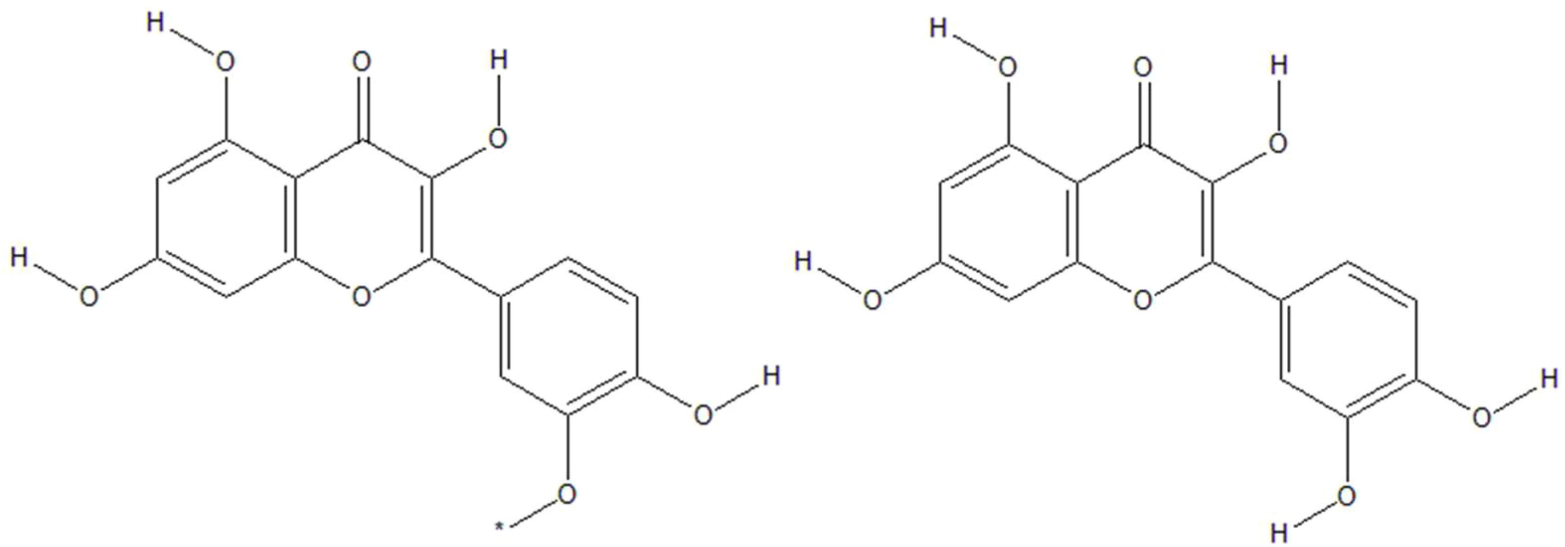
Sea buckthorn polyphenolic flavonoids isorhamnetin (left) and quercetin (right).

Together with leaves, sea buckthorn berries are rich in a variety of vitamins and other physiologically active components, including up to 106 nutraceutical and 74 bioactive chemicals (13) or even up to 190 bioactive compounds (4). Different parts of the plant contain phenols, carotenoids (lycopene, carotene, lutein, and zeaxanthin), flavonoids (isorhamnetin, quercetin, glycosides, and kaempferol), tocopherols, sterols (4, 13–15), polyunsaturated fatty acids, minerals, vitamins, omega 3, 6, 9 and rare omega 7 fatty acids (4), and dietary fibers (16). The oil derived from sea buckthorn seed is the only natural oil that contains a 1:1 ratio of omega 3 and omega 6 fatty acids (linolenic and linoleic acids) and has β-sitosterol as primary phytosterol (16). Berries are an excellent supply of vital polyunsaturated fatty acids, sugars, and tocopherols, while leaves are a good source of polyphenols (17, 18). *H. rhamnoides L. subsp. yunnanensis* (Yunnanensis)*, H. rhamnoides L. subsp. mongolica* (Mongolica)*, H. rhamnoides L. subsp. turkestanica* (Turkestanica) *and H. rhamnoides L. subsp. sinensis* are four different subspecies of sea buckthorn that have had their phytochemical compositions studied. *H. rhamnoides L. subsp. yunnanensis* has the largest cellular antioxidant and antiproliferative characteristics, whereas *sinensis* subspecies has the highest total phenolic content and related total antioxidant activity (19). Total flavonoid concentration of sea buckthorn is around 23 mg quercetin equivalent/g dried extract, and total polyphenol content is about 46 mg gallic acid equivalent/g dried extract (20). However, the bioactive content of berries is also affected by age, fruit size, climate, geographic location, and extraction process (21). Zheng et al. (22) found a variety of beneficial chemicals in these berries, including oleanolic acid, 19-alpha-hydroxy ursolic acid, succinic acid, ursolic acid, 5-hydroxymethyl-2-furancarbox-aldehyde, octacosanoic acid, palmitic acid, hippophae cerebroside, and 1-O-hexadecanolenin. Recently, a number of phytoprostanes, phytofurans, tocopherols, tocotrienols, carotenoids, and free amino acids have been detected in sea buckthorn berry juice (23). Berries contain high amounts of polysaccharides (18) and dietary fibers (7, 16). Some bioactive phenolic components, including quercetin-3-O-galactoside, quercetin-3-O-glucoside, kaempferol, and isorhamnetin (24, 25), as well as flavonol glycosides (di- and tri-glycosides) (15) have been detected in leaf extracts. Six compounds from sea buckthorn leaf extract have been isolated previously: kaempferol-3-O- β-α-(6’’-O-coumaryl) glycoside, 1-feruloyl-β-α-glucopyranoside, isorhamnetin-3-O-glucoside, quercetin-3-O-β-α-glucopyranoside, quercetin-3-O-β-α-glucopyranosyl-7-O-α-l-rhamnopyranoside, and isorhamnetin-3-O-rutinoside (26). Tannin fractions from leaves have been separated, and the main components are hydrolyzable gallo- and ellagi-tannins of the monomeric type: strictinin, isostrictinin, casuarinin, and casuarictin (25). Data show that sea buckthorn is a rich source of several biologically active compounds that may be helpful to health and effective in the prevention and treatment of a variety of illnesses (27). As mentioned above, key sea buckthorn polyphenolic flavonoids include isorhamnetin and quercetin (**Figure 1**).

## 3 Physiological and therapeutic actions

In recent years, research has shown that sea buckthorn can help with illness prevention and healing, including viral infections and cancer (15, 28) owing to its antioxidant (29, 30), anti-inflammatory (31), antiviral (32), antimicrobial (24, 30, 33), and antibacterial (34, 35) properties. Cardioprotective, anti-atherogenic, hepatoprotective, hypolipidemic (29, 36, 37), dermatological (4), antiproliferative (20), and anticancer (e.g., colon, liver, lung, cervical, ovarian, and breast cancer cells) effects have been reported, too (6, 15, 38). Proanthocyanidins, curcumin, and resveratrol have been demonstrated to have considerable advantages in cancer chemoprevention and radiotherapy (39). Similarly, kaempferol has been shown to suppress the growth of breast cancer (40). A higher dietary intake of phenolic substances, particularly flavonoids, and procyanidins, has been linked to a decreased risk of cancer (41). Aurolognans, bioactive constituents of sea buckthorn, have been linked with hepatoprotective, hypolipidemic, and anti-obesity effects (37). Moreover, anti-inflammatory action of this plant could be due to the presence of triterpenes – oleanolic, asiatic, and maslinic acids (42). **Table 1** summarizes the physiological and therapeutic activities of sea buckthorn preparations through *in vivo* and *in vitro* experimentations on several experimental models.

**Table 1:** Physiological and therapeutic actions of sea buckthorn preparations.

Action (s)	Preparation	Experimental model	Results	Reference(s)
Anti-inflammatory	Ethanolic leaf extract	Human keratinocytes cell line HaCaT, human monocytic leukemia cell line THP-1	Inhibition of tumor necrosis factor α (TNF α) and intercellular adhesion molecule 1 expression by casuarinin present in sea buckthorn; decrease in TNF α-induced pro-inflammatory mediators, such as interleukin 6, interleukin 1β, interleukin 8, monocyte chemoattractant protein-1	(31)
	Myricetin (a flavonoid from sea buckthorn)	Rats with high-fat-diet	Reducing inflammation by regulating butyric acid producing intestinal microorganisms and protecting intestinal barrier function	(43)
	Sea buckthorn extract	Rats with high-fat-diet	Promotion of ZO-1 and occludin mRNA expression in intestinal tight junction proteins; repair of intestinal mucosa; anti-inflammatory role in inhibiting the signal pathway of NOD-like receptor	(44)
Antioxidant	Aqueous seed extract	Liposome model system, *Listeria monocytogenes, Yersinia enterocolitica*	Good antioxidant effect in different assay systems (reducing power, DPPH assay and liposome model system).	(33)
	Fermented sea buckthorn juice	H_2_O_2_-treated C_2_C_12_ cells	Increase in intracellular SOD and glutathione peroxidase (GSH-Px) activity; decrease in ROS content, catalase (CAT) activity, and malondialdehyde (MDA) content	(30)
	Aqueous and hydroalcoholic leaf extract	Baby hamster kidney cell line 21 BHK-21, *Bacillus cereus, Pseudomonas aeruginosa, Staphylococcus aureus, Enterococcus faecalis, Escherichia coli*	Potent antioxidant activity determined by ABTS, DPPH and FRAP assays; cytoprotective activity against hydrogen peroxide, hypoxanthine-xanthine oxidase induced cell damage; growth inhibition against bacteria.	(24)
Antiviral	Ethanolic leaf extract	Dengue virus type-2 infected human peripheral blood mononuclear cells	Maintaining the cell viability of Dengue-infected cells, decrease in the TNF α and increase in the interferon γ production in Dengue-infected cells.	(32)
	Sea buckthorn leaf extract	MDCK (Madin-Darby Canine Kidney) cells infected with influenza viruses A/Victoria, A/PR, B/Lee and B/Maryland	Extreme anti-influenza activity; flavonols did not interact directly with influenza viral particles, and inhibited initial stage of virus replication only	(15)
	Sea buckthorn DMSO extract	HSV-2 infected Vero cells	Dose dependent inhibitory effect against HSV-2 virus	(45)
Antibacterial	Phenol rich fraction from leaves	*Escherichia coli*, *Salmonella typhi*, *Shigella dysenteriae*, *Streptococcus pneumoniae* and *Staphylococcus aureus*	Broad spectrum antibacterial effect by growth inhibition of certain medically important bacterial species.	(34)
	Chitosan extracted from sea buckthorn leaves	*Staphylococcus aureus, Escherichia coli, Salmonella typhimurium, Listeria monocytogenes*	Enhanced antibacterial properties	(30)
	Sea buckthorn seeds	*Escherichia coli, Staphylococcus aureus, Salmonella, Pseudomonas aeruginosa, Bacillus subtilis*	Potential promotion of food preservation by sea buckthorn seed polyphenols	(46)
Anticancer	Ethyl acetate and ethanol:water sea buckthorn extracts	Co-cultured human intestinal epithelial cell line Caco-2, human hepatocyte carcinoma cell line Hep G2	Dose-dependent antiproliferative effect by inhibition of cancer cell proliferation.	(6)
	Copper nanoparticles synthesized from stem extracts of sea buckthorn	HeLa cells	Concentration-dependent reduction in cell viability (as measured by MTT assay), and apoptotic activity (as detected by ROS production)	(47)
	Sea buckthorn leaf extract	Lung cancer cells NCL-H1299, human ovarian cancer cells HeLa and SKOV, and cervix cancer cells Caski	Fascinatingly higher and wider range of cytotoxic activities against lung, ovarian, and cervical cancer cells	(15)
Hepatoprotective	Ethanolic extract of berries	Male mice C57BL/6	Protective effect against acetaminophen (APAP)-induced hepatotoxicity associated with the activation of the Nrf-2/HO-1-SOD-2 signaling pathway; suppression of APAP-induced increase in the ratio of B-cell lymphoma protein 2-associated X.	(13)
	Ethanolic extract of sea buckthorn berries	Pathogen-free Kunming mouse	Sea buckthorn flavonoids significantly reduced the weight, liver fat accumulation, and serum triglyceride level of obese mice induced by high-fat diet and inhibited the chronic inflammatory reaction caused by obesity	(48)
	Sea buckthorn berry seed oil (SBO)	BALB/c mice	Protective effect of SBO against cyclophosphamide -induced liver damage, which reflected its antioxidant properties	(49)

### 3.1 Protective role against cardiovascular diseases, metabolic syndrome, and obesity

The physicochemical and functional features of sea buckthorn berry pomace powder (PP) justify its usage as a fiber-rich dietary additive (16). PP had strong hydration qualities in addition to having a high protein content (21.09 g/100 g) such as 4.24 g/g and 9.98 mL/g of water-holding capacity and swelling capacity, respectively. The functional potential of the tested PP was determined by its *in vitro* hypoglycemic and hypolipidemic qualities, which were shown to be comparable to and, in some cases, superior to those of other dietary fiber powders made from by-products of the processing of fruits and vegetables. Berry PP had a cholesterol-binding capacity of 21.11 to 23.13 mg/g (16). Traditionally, sea buckthorn berries also serve as a Chinese medicine with multiple bioactivities (18). A recent bioassay-guided investigation applied to seek the hepatoprotective and hypolipidemic ingredients has been able to isolate three new (10 → 10’’)-biauronlignans (1–3), three new 10-(4’’-hydroxy-benzyl)-auronlignans (4–6), three new 10-O-β-D-glucopyranosyl-auronlignans (7–9), and eleven known auronlignan derivatives (10–20). Their structures have been established using lengthy and thorough infrared (IR), ultraviolet/visible (UV/Vis), nuclear magnetic resonance (NMR), and mass spectroscopy (MS) spectrum investigations, and these results have been compared with the published references. While compounds 2, 5, 8, and 12 displayed mild pancreatic lipase activity inhibition and reduced the moderately FFA-induced lipid accumulation in HepG2 liver cells, compounds 1, 4, 7, 11, 15, and 19 demonstrated moderate hepatoprotective activities against the damage in acetaminophen-induced HepG2 cells (37). In addition, structural data of a homogeneous polysaccharide from sea buckthorn (SBP-1-A) has recently been described, and it was discovered that SBP-1-A has a backbone of around 3,4).-β-l-Rhap-(1 → 4)-α-d-GalAp-(1 → with side chains made up of α-l-Araf, β-d-Galp, β-d-Glcp, and α-d-Glcp, of which the arabinose, glucose, and galactose residues have been identified as the primary monosaccharide compositions with a percentage surpassing 92%. Furthermore, the protein-free polysaccharide fraction (SBP-1) obtained after isolation of crude SBP showed an outstanding anti-obesity effect. According to the findings, consuming SBP-1 might increase the expression of peroxisome proliferator-activated receptor gamma coactivator 1 (PGC1α), uncoupling protein 1 (UCP-1), and PR domain containing 16 (PRDM 16) in adipocytes, activate brown adipocytes, and boost thermogenesis, which would prevent fat buildup and weight gain. It is crucial to remember that the type of preparation, its chemical composition, and its concentration all appear to have an impact on how sea buckthorn preparations affect hemostasis. The sea buckthorn preparations seem to be excellent regulators of hemostasis, particularly blood platelet function, due to their high phytochemical contents, notably phenolic components. Additionally, it is still uncertain how much of these preparations should be used for prophylaxis and therapy, and recommendations for using sea buckthorn preparations are frequently based on sparse clinical investigations. Thus, more randomized clinical studies with bigger samples are required, particularly those including healthy volunteers and those with the greatest cardiovascular risk factors. Additionally, the effects of several sea buckthorn components on hemostasis, including fibrinolysis and coagulation systems as well as blood platelet activities, should be studied in these trials. Since there is currently no reliable information about the anti-hemorrhagic effectiveness of sea buckthorn preparations in either people or animals, it is also crucial to investigate the role of various sea buckthorn products in the prevention and treatment of cardiovascular disorders (50). Recently, phytoprostanes in sea buckthorn juices have been discovered, and their quantities are highly connected with the capacity to reduce inflammation through inhibition of the 15-lipoxygenase enzyme (23). Due to the presence of possible inhibitors of α-amylase, α-glucosidase (tocopherols, tocotrienols, and certain amino acids), and pancreatic lipase (xanthophylls), sea buckthorn juice can be an intriguing anti-diabetic and anti-obesity diet. Juices act more effectively in lowering neurological alterations due to the presence of phytoprostanes, phytofurans, tocopherols, tocotrienols, and amino acids, making them possible anti-aging agents in the prevention of Alzheimer’s disease, the most prevalent kind of dementia. Juice from sea buckthorn may be crucial in the body’s fight against diseases brought on by free radical assault (23). Sea buckthorn insoluble dietary fiber (IDF) can be modified to increase its *in vitro* hypoglycemic capacity. Examples of these modifications include IDF, milled insoluble dietary fiber, and co-modified insoluble dietary fiber. Ball milling, as well as ball milling coupled with cellulose treatment reportedly enhanced the characteristics of IDF which provide a foundation for the extensive utilization of sea buckthorn resources (27).

### 3.2 Anti-inflammatory properties

Maslinic acid functions via the nuclear factor kappa light chain enhancer of activated B cells (NF-κB) and erythroid 2-related factor 2 (Nrf2) signaling pathways, whereas oleanolic and asiatic acids act via the NF-κB, mitogen-activated protein kinase (MAPK), and Nrf2 signaling pathways to exert anti-inflammatory effects on macrophages. These three substances can be employed as natural anti-inflammatory dietary supplements because they exhibited specific inhibitory effects on the LPS-induced inflammatory response *in vitro*. However, more research is necessary, including *in vivo* investigations, to encourage the usage of sea buckthorn-derived products (42). The active components of sea buckthorn that are responsible for the biological effects haven’t yet been fully identified. The flavonoids quercetin and isorhamnetin, a 3′-O-methylated metabolite of quercetin, are thought to be principally in charge. There is proof that isorhamnetin can prevent cells from proliferating (42), promote apoptosis and mitigate tumor development (51–53), suppress inflammatory processes (33, 42, 54), improve cognitive functions (54), and affect numerous metabolic processes (33). The anti-cancer (29, 55), cardioprotective (56), and anti-obesity (57) effects of quercetin have been reported, too. Presence of several flavonoids including isorhamnetin (54, 58), auronlignan (18), polysaccharides (37, 59), and dietary fibers (16, 27) indicate towards the anti-cholesterol and anti-obesity effects of sea buckthorn. Thus, a number of physiological and pathological processes can be targeted by sea buckthorn and its bioactive compounds. Nevertheless, a majority of the studies on sea buckhorn action were performed for medicinal purposes in pathological conditions, including on cancer cells. Therefore, obtained information could potentially be helpful for management of tumors, but the biological value of the information is limited due to the fact that whether and how the dietary consumption of sea buckwheat could affect healthy organism and its cells. Even the applicability of sea buckthorn for treatment of cancer is yet to be sufficiently demonstrated by clinical trials. Furthermore, sea buckthorn constituents responsible for the effect of the whole plant remains to be identified. The molecules, which might be responsible for sea buckhorn effect have been hypothesized on the basis of their presence in the plant and the similarity of their as well as whole plant effects. But there isn’t a single thorough experiment that has compared these impacts. Furthermore, there is no conclusive evidence of a functional connection from the similarities of the effects. Therefore, extensive research is needed to identify the components of sea buckthorn that are responsible for its biological and therapeutic effects.

## 4 Mechanism of action

### 4.1 Mechanism of action of sea buckthorn

Although the evidence for each of these processes is weak and the interactions between these mechanisms are poorly understood, extracellular and intracellular modes of action of sea buckthorn and its constituents on cells have been postulated. Nevertheless, it has been proposed that the ability of sea buckthorn to prevent and to treat infections and cancer (15) as mentioned previously can mainly be due to its antioxidant (29), anti-inflammatory (31), antiviral (32), antimicrobial (24, 33), and antibacterial (34, 35) properties. Numerous clinical disorders, including allergies, cancer, and many others, are mostly driven by inflammation. By stimulating Nrf2-dependent pathways, sea buckthorn’s anti-inflammatory effects may be mediated (60). An effective anti-inflammatory target, the heme oxygenase-1 (HO-1) axis, is known to be regulated in part by Nrf2. Nrf2 is essential for regulating the production of antioxidant genes, which in turn have anti-inflammatory effects (60). The protective action of sea buckthorn polysaccharide is linked to the activation of the Nrf-2/HO-1-SOD-2 signaling pathway (36). Recent investigations revealed a relationship between the production of additional inflammatory mediators including the NF-κB pathway and macrophage metabolism and the Nrf2/antioxidant response element system (60). It’s interesting to note that a sea buckthorn polysaccharide has been shown to protect the liver against acetaminophen (APAP)-induced liver damage in rats. Enzymes like alanine aminotransferase (ALT) and aspartate aminotransferase (AST) have been able to be reduced by it (36). The antioxidant qualities of sea buckthorn’s components have been used to describe a variety of its therapeutic benefits. In the liver, brain, and plasma, for instance, sea buckthorn supplementation elevated glutathione (GSH) and GSH-Px levels and the production of nitric oxide (NO) and the inducible nitric oxide synthase (iNOS), which was linked to lessened liver damage (36) and oxidative and nitrosative stress in liver and brain of rats (61). Antioxidant chemicals, especially phenolic components such as flavonoids kaempferol, isorhamnetin, and quercetin, are responsible for sea buckthorn’s antitumor action. These flavonoids defend against oxidative stress, which can cause cancer and genetic alterations in cells (50, 62). The formation of ROS was reduced along with the reduction in glioma cell viability after sea buckthorn extract treatment, at the very least (26). Masoodi et al. (63) has proposed that sea buckthorn reduces the production of a certain antigen and inhibits cellular growth in prostate cancer cells. Additionally, rat glioma cells’ fast multiplication was suppressed by sea buckthorn leaf extract, which is thought to have done so via causing the first stages of cell death. The increased expression of B-cell lymphoma 2/Bcl-2-associated X protein (Bcl-2/Bax) and acetaminophen-induced inhibition of c-Jun N-terminal kinase phosphorylation are further signs of sea buckthorn’s impact on cytoplasmic apoptosis (36). The pro-apoptotic Bax gene expression was increased by sea buckthorn extracts, and its localization, accumulation, and translocation in the nuclei were all encouraged (26). However, as demonstrated in human retinoblastoma cells, the quercetin-induced rise in cytochrome c levels together with the activation of caspase-3 and caspase-9 results in apoptosis in cancer cells (64). Additionally, it was shown previously that buckthorn procyanidins might cause cell death in a dose-dependent way (65). It is possible that these procyanidins might block intracellular fatty acid synthase (FAS) activity and cause human breast cancer cells to undergo apoptosis. At least, sea buckthorn procyanidins were discovered to restrict the proliferation of cancer cells, and FAS is a critical enzyme for *de novo* long-chain fatty acid production, which is present in high amounts in cancer cells (65). Several flavonoids (especially isorhamnetin) affecting a number of enzymes regulating fat synthesis and metabolism can be responsible for their hypolipidemic, cholesterol-lowering, and anti-obesity effects (58). In addition, the anti-obesity effects of sea buckthorn polysaccharides could be due to their stimulatory action on brown adipose tissue and thermogenesis inducing its “burning” (37). Finally, sea buckthorn’s anti-diabetic and anti-obesity effects can be attributed to the ability of its dietary fibers to reduce glucose production and metabolism via suppression of glucose adsorption, glucose diffusion inhibition, starch digestion inhibition, starch pasting interference, and α-amylase activity (27). Androgen receptors are the next potential mediator of sea buckthorn’s actions on tissues that are dependent on hormones. These receptors control the expression of androgen-responsive genes through ligand-dependent transcription factors. The target androgen responsive genes cannot be activated, and prostate cancer growth cannot be stopped if the androgen receptor is somehow kept in the cytoplasm and its shuttling into the nucleus is blocked. Prostate cancer cells’ androgen receptors have been shown to be affected by the administration of sea buckthorn leaf extracts, which was correlated with the suppression of genes that respond to androgens, cellular growth, and survival of these cells (63). The beneficial effects of sea buckthorn on spermatogenesis may be due to its effect on androgen receptors. By increasing spermatogonia proliferation, stem cell survival, and lowering sperm abnormalities, sea buckthorn therapy enhanced spermatogenesis and had a protective effect against the negative effects of gamma radiation (66). Available literature demonstrates a number of signaling molecules and mechanisms mediating sea buckthorn’s actions on various targets in the organism and/or their pathologies at a cellular level. Some of their mediators could be specific for particular target cells or organs (e.g., steroid hormones for steroid-dependent processes). Other mediators could be more pervasive, such as those that influence cell proliferation, apoptosis, and oxidative processes. Additionally, although they haven’t been fully explored yet, functional hierarchy linkages between the mediators of sea buckthorn’s effects are feasible. Last but not least, studies of the effects of sea buckthorn and its mediators have primarily used cell cultures. As a result, appropriate *in vivo* research should be used to confirm the findings gained using such models.

### 4.2 Mechanism of action of selected sea buckthorn constituents – isorhamnetin and quercetin

**Figure 2:**
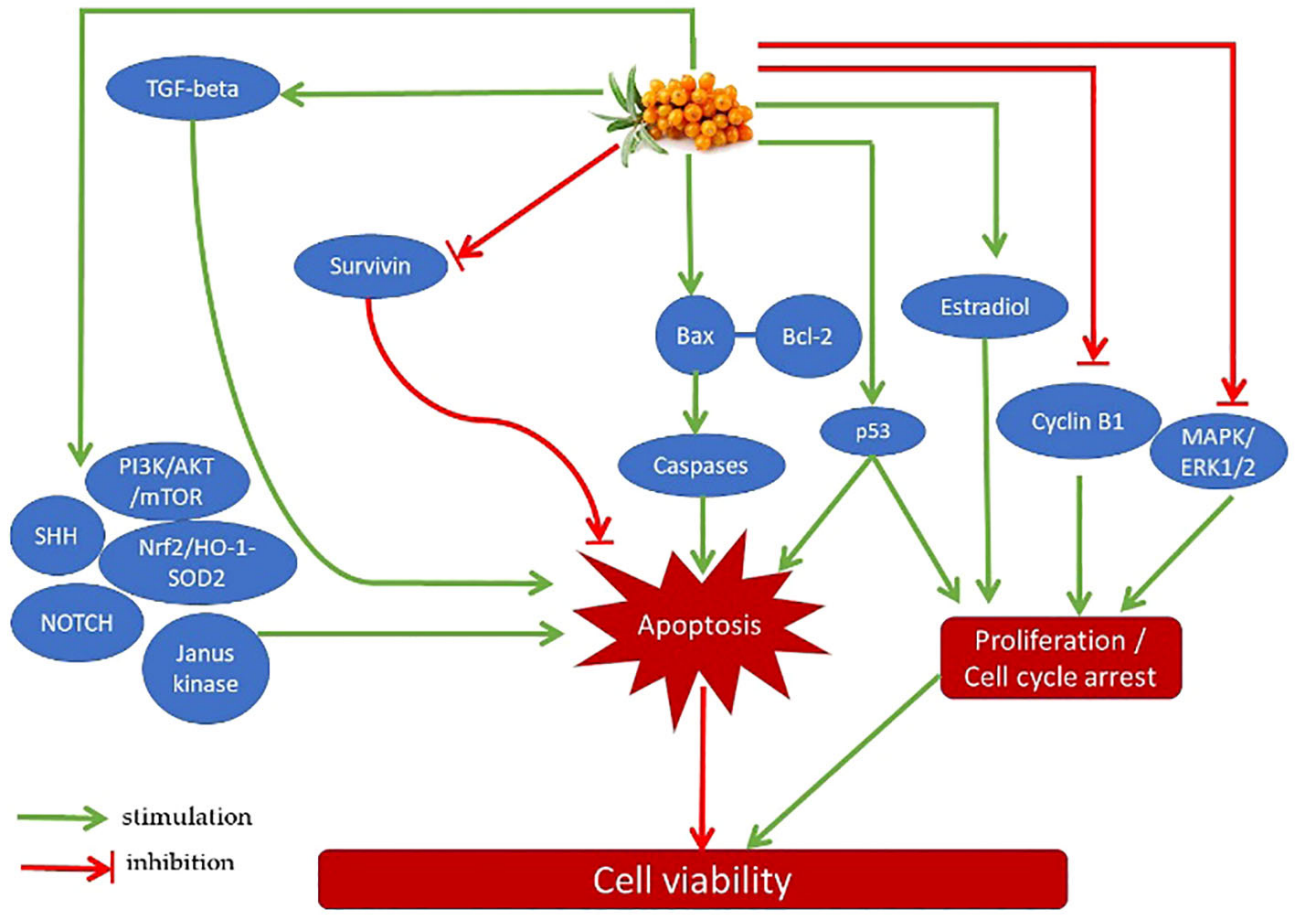
Possible mechanisms of action of sea buckthorn and its bioactive components on cancer cells.

One of the most potent active components in sea buckthorn berries is isorhamnetin, a 3′-O-methylated metabolite of quercetin that has a wide range of pharmacological effects, including anti-cancer ones. The modulation of PI3K/AKT/PKB, NF-κB, MAPK, and other signaling pathways, as well as the production of associated cytokines and protein kinases involved in controlling cell apoptosis and proliferation, are all part of the mechanisms of action (53, 54). Through the activation of apoptotic genes and apoptosis and the downregulation of oncogenes, isorhamnetin can inhibit the development of cancer. Isorhamnetin has also been demonstrated to inhibit the PI3K-AKT-mTOR pathway (phosphatidylinositol 3-kinase, protein kinase B, and the mammalian target of rapamycin), which in turn inhibits the proliferation of cancer cells by inducing cell cycle arrest at the G2/M phase. Additionally, isorhamnetin can increase the production of cyclin B1 protein while decreasing the phosphorylation levels of AKT, phosph-p70S6 kinase, and phosph-4E-BP1 proteins (52). Additionally, isorhamnetin can enhance liver and kidney functioning by lowering blood levels of urea nitrogen, AST, and ALT. Additionally, by preventing the dimerization of the toll-like receptor 4, isorhamnetin can reduce infection-induced liver and kidney inflammation as well as inflammation-induced cell death (33). Some physiological actions of isorhamnetin could be mediated by an interplay of several intracellular signaling pathways. For example, isorhamnetin can mitigate the adverse effect of obesity on cognitive functions by suppression of neuroinflammation via downregulation of MAPK- and NFkB-dependent pathways (54). Quercetin is another ingredient that may contribute to the benefits of sea buckthorn. Quercetin’s ability to influence intracellular signaling pathways that regulate cell proliferation and apoptosis may be the cause of its anti-cancer effects (29, 55, 67). It can cause cell cycle arrest through suppression of its promoters cyclin B1 and MAPK/ERK1/2 and activation of transcription factor p53. It can also prolong DNA repair and promote apoptosis through inhibition of survivin, activation of transforming growth factor-β (TGF-β), PI3K/AKT/mTOR, Wnt/-catenin, NOTCH, sonic hedgehog signaling pathway (SHH), Janus kinas Additionally, quercetin can inhibit tumorigenesis by controlling VEGF and its receptors, which are the factors that stimulate tumor vascularization (55). The presence of quercetin, tannins, and other polyphenolic flavonoids in sea buckthorn extract, as well as their radical scavenging and anti-inflammatory properties, may be responsible for the protection of spermatozoa (66), and cardiomyocytes (56, 57). Some possible mechanisms of action of sea buckthorn and its bioactive components on cancer cells are summarized in **Figure 2**. In fine, the literature concerning the mechanisms and/or mediators of action of sea buckhorn compounds isorhamnetin and quercetin indicate an existence of multiple pathways of these molecules on target cells. The key mechanisms of their action (e.g., related to cell proliferation, apoptosis, or oxidative stress) are like mechanisms of sea buckthorn whole plant’s effects. Nevertheless, it remains to be investigated whether the effects of the whole plant and its mechanisms

of action could be explained by the presence of only isorhamnetin and quercetin, or other constituents and their mechanisms of action could be involved in mediating sea buckthorn’s effects. Furthermore, the data concerning these mediators, which were obtained predominantly by *in vitro* experiments, require verification by corresponding *in vivo* studies.

## 5 Effect on female reproductive processes

### 5.1 Ovary cancer preventive effects of sea buckthorn

There is no evidence accessible in scientific databases about the effects of entire sea buckthorn on ovarian functions. Dietary sea buckthorn oil did not influence bovine ovarian folliculogenesis, oocyte quality, or embryo developmental ability (68). Nonetheless, some physiologically active sea buckthorn elements have been shown to influence female reproductive processes. However, the direct impact of quercetin on fundamental ovarian cell processes (proliferation, apoptosis, and hormone release) may vary depending on the species (69). Quercetin also inhibits the development of human metastatic ovarian cancer cells and affects the intrinsic apoptotic mechanism (70–73). Isorhamnetin, another sea buckthorn component, can stimulate ovarian cell proliferation and estrogen release (52, 70), and suppress estrogen-dependent ovarian cancer development (70, 73). Other physiologically active components of sea buckhorn have also been proven to be advantageous to ovarian cancer cells. Apigenin, myricetin, and luteolin have been shown to cause apoptosis, decrease cell proliferation, limit cell invasion, and stop the cell cycle of ovarian cancer. Furthermore, apigenin, myricetin, and luteolin have been recommended as prospective ovarian cancer preventative and adjuvant therapy options (70, 74). Another sea buckthorn ingredient that can inhibit ovarian cancerogenesis is kaempherol. In cultivated ovarian cancer cells, it can at least trigger apoptosis and halt the cell cycle (70, 73, 75–77). Furthermore, kaempherol can inhibit angiogenesis in ovarian tumors (76). As a result, sea buckthorn components may influence ovarian cell proliferation, death, and hormone release. In ovarian cancer cells, on the other hand, sea buckthorn components can cause apoptosis, decrease cell proliferation, and stop the cell cycle. Nonetheless, the relevance of the collected data is restricted by the fact that the claimed effects on ovarian functions are primarily the product of *in vitro* investigations, with majority of these tests being done on ovarian cancer cells rather than healthy cells. There is a need for more information on the entire sea buckthorn’s activity on ovarian functions (including dysfunctions and malignant transformation) both *in vitro* and *in vivo*.

### 5.2 Effect on vagina and uterus

Traditionally, sea buckthorn has been used to treat gynecological diseases such as uterine inflammation and endometriosis. Its oil helps reduce the symptoms of endometriosis and uterine inflammation. These effects might be linked to the carotenoid, sterol, and hypericin content of the plant (55). Consumption of sea buckthorn extract or oil may also be beneficial in the avoidance of vaginal difficulties during menopause, which is associated with vaginal atrophy and the thinning and drying of the vaginal mucosa. Menopausal women who used sea buckthorn oil had better vaginal epithelial integrity and a higher vaginal health score. It has been proposed as an alternative to estrogen replacement therapy for postmenopausal women’s vaginal health (78). Furthermore, a novel vaginal gel containing sea buckthorn oil (Meclon Idra Alfasigma) has recently been registered. It appears to be a viable option as a local agent for alleviating symptoms of vulvovaginal atrophy (vaginal dryness, itching, and burning feeling) and enhancing sexual function in postmenopausal women (79). Sea buckthorn also has a high concentration of vitamins C and E. Infertile or subfertile women undergoing controlled ovarian stimulation may benefit from vitamin C and E supplementation in terms of uterine features, endometrial thickness, and endometrial blood flow. Furthermore, the antioxidant and anticoagulant properties of vitamins C and E are assumed to be responsible for the increase in fertility (80). In contrast to its effect on the ovary, sea buckthorn, and its components have been shown to have a therapeutic effect on the management of gynecological problems such as uterine inflammation, endometriosis, and signs of vulvovaginal atrophy in postmenopausal women. We summarized the important effects in **Table 2**. However, the effect of sea buckthorn on the healthy vagina and uterus has not been thoroughly established. Furthermore, the components of sea buckthorn that affect these organs are mainly unknown. Even the role of vitamins in mediating the benefits of sea buckthorn is more or less theoretical and requires scientific validation.

**Table 2:** Physiological and therapeutic actions of sea buckthorn and its constituencies relating to female reproductive processes.

Therapeutic action(s)	Preparation	Experimental model	Reference(s)
*In vivo* studies			
Prevention of vaginal complications during menopause	Sea buckthorn extract/oil (Three months, during which subjects consumed 3 g of sea buckthorn or placebo oil daily)	Menopausal women	(78)
Improving sexual function in postmenopausal women	Sea buckthorn oil (Active vaginal gel or placebo was applied for 14 days and then twice a week for 90 consecutive days)	Menopausal women	(79)
Can improve uterine characteristics, endometrial thickness, and endometrial blood flow	Sea buckthorn [2 years combined therapy of clomiphine citrate (from day 2-6 of the cycle) and vitamins E(400mg) and C(500 mg) (ndash;from day 1-14 days of the cycle)]	Infertile and subfertile women	(80)
Cell line studies			
Suppresson of estrogen-dependent ovarian cancer development	Isorhamnetin and quercetin DMSO solution	Human ovarian granulosa-like KGN cells	(70)
Promoting ovarian cell proliferation, estrogen release	Isorhamnetin DMSO solution	Human ovarian granulosa-like KGN cells	(70)
Inducing apoptosis and blocking cell cycle in cultured ovarian cancer cells	Kaempherol DMSO solution	Human breast cancer MCF-7 and MDA-MB-453 cells	(75)
Suppression of angiogenesis in ovarian tumor	Kaempherol DMSO solution	Triple-negative breast cancer cells (TNBC)	(76)

## 6 Mechanism of action on female reproductive processes

There is inadequate information to support the mechanism of sea buckthorn’s impacts on female reproductive systems. Nonetheless, existing evidence allows us to sketch some of the processes and mediators of sea buckthorn or its active ingredients in the female reproductive system. Sea buckthorn oil has been shown to reverse endometriosis in rat. The therapy lowered the levels of inflammatory cytokines (inflammation markers and promoters) and VEGF (angiogenesis markers and promoters) (55). Therefore, cytokines and VEGF could be extracellular mediators of the curative action of sea buckthorn on endometriosis. Imran et al. (76) also hypothesized that kaempferol would reduce tumor development and angiogenesis by lowering VEGF expression via hypoxia-inducible factor 1α (HIF-1α), a physiological activator of VEGF synthesis. Some sea buckthorn flavonoids have also been shown to operate on ovarian cells via intracellular regulators of proliferation and death. For example, quercetin promotes caspase-3 expression, which may result in DNA fragmentation and apoptosis. In addition, quercetin has been demonstrated to reduce the expression of anti-apoptotic proteins while increasing the synthesis of pro-apoptotic proteins in a variety of cancer cell lines, including ovarian cancer (71, 72). Isorhamnetin has the potential to influence ovarian cancer cell proliferation and apoptosis by targeting intracellular PI3K/Akt signaling pathway promoters of the cell cycle (cyclins) and apoptosis (Bax, Bcl, and cytochrome) (52, 77). Kampherol has been shown to enhance the production of morphological indications of apoptosis (membrane blebbing) and the accumulation of apoptotic intracellular markers and promoters (caspases 3, 8, and 9, as well as Bax) while decreasing the expression of anti-apoptotic Bcl-2. Furthermore, kaempferol caused cell cycle arrest at the G0/G1 checkpoint, as well as inhibition of cyclin B1 and Cdc2 expression (75). Imran et al. (76) suggested that kaempferol can induce apoptosis and cell cycle arrest at the G2/M phase via upregulation of checkpoint kinase 2/cell division cycle 25C/cyclin-dependent kinase 2 (Chk2/Cdc25C/Cdc2), receptors DR5 and DR4, c-Jun N-terminal kinase (JNK), C/EBP homologous protein (CHOP), p38, p21, the extracellular signal-regulated kinase 1/2 (ERK1/2) proteins, caspase-3, -7, -8, Bad, Bax, and p53 proteins. The ability of sea buckthorn (50) and isorhamnetin (52) to affect ROS, the known promoters of apoptosis, indicates that this sea buckthorn flavonoid can impact ovarian cell viability and result in events impacting oxidative stress. Finally, sea buckhorn constituents isorhamnetin (52, 70) and quercetin (69, 81) can affect the production of estrogens and other ovarian hormones, which are considered as key regulators of ovarian functions and female reproduction and fecundity (81). This fact indicates that sea buckthorn might impact reproductive processes through hormonal mechanisms, too. The few relevant published findings suggest that sea buckthorn may be useful in the treatment of endometriosis by influencing extracellular regulators of inflammatory processes such as cytokines and VEGF. However, the intracellular mediators of this therapeutic activity still need to be identified and verified. It is uncertain whether this plant or its active ingredients have an effect on the healthy vagina and uterus. More is known about the mechanisms/mediators of sea buckthorn and its components’ impact on ovarian cells. Sea buckthorn flavonoids have been shown to suppress ovarian cancer cells by downregulating VEGF, anti-apoptotic proteins, upregulating pro-apoptotic proteins, suppressing the cell cycle at various checkpoints, p-AKT, and inducing oxidative and endoplasmic reticulum stress and autophagy. Hormones may also play a role in modulating the effects of plant constituents’ isorhamnetin and quercetin on female reproductive organs. However, it should be emphasized that the mediators and processes of sea buckthorn or its components are postulated primarily on the basis of indirect evidence - since these regulators have been altered following the administration of the plant or its constituents. More direct experimental data is needed to understand the functional interrelationships between plant compounds and reproductive process regulators. When compared to known mediators of its effects on non-reproductive processes, the number of recognized mediators of sea buckthorn on female reproductive organs is small. More research would very certainly add to the list of mediators of sea buckthorn’s physiological and therapeutic effects on female reproductive systems. Although hierarchical interrelationships between numerous mediators of sea buckthorn’s activities on the ovary, vagina, and uterus are plausible, they have yet to be thoroughly investigated.

## 7 Potential for application in reproduction

Sea buckthorn is being studied as a functional food as well as a herbal medication for animal and human health, including the treatment of numerous female reproductive diseases. Although there are some publications on the effects of sea buckthorn chemicals quercetin and isorhamnetin on healthy ovarian cells, it is uncertain if sea buckthorn extract or its constituents could be effective in influencing healthy female reproductive processes. More data, however, is available on the use of sea buckthorn and its components to prevent and/or perhaps treat ovarian cancer. Flavonoids found in sea buckthorn can decrease cancer cell proliferation, cause apoptosis, prevent cell cycle arrest, and slow tumor development. This might point to the possible use of sea buckthorn flavonoids in the prevention and treatment of ovarian cancer.

Furthermore, ovarian cancer is linked to other gynecological diseases such as endometriosis (82). The potential use of sea buckthorn and its active ingredients in the treatment of gynecological problems such as uterine inflammation, endometriosis, and symptoms of vulvovaginal atrophy in postmenopausal women has been proven (82). No toxicity of sea buckthorn berries (50) or sea buckthorn berry oil (8) has been reported including no treatment-related maternal toxicity or embryotoxicity (8). According to the data, sea buckthorn products can be used as functional foods, nutritional supplements, and medicines. However, it cannot be ruled out that extracted and purified sea buckthorn compounds/molecules might be used in place of dietary sea buckthorn or its extract. Although such a substitute may be more expensive, the dose and ingredients may be easier to define, and the biological and/or therapeutic efficiency may be greater and more predictive than the raw plant product. Taken together, the available evidence on sea buckthorn’s beneficial effects suggests that it has potential therapeutic applications in phytotherapy of cancer, endometriosis, and/or other reproductive dysfunctions.

## 8 Conclusions and possible direction of future studies

Sea buckthorn elements appear to alter healthy ovarian cell proliferation, death, and hormone release, as well as decrease ovarian cancer (by triggering ovarian cancer cell apoptosis and autophagy, decreasing cell growth, invasion, and halting the cell cycle). Furthermore, sea buckthorn and its bioactive ingredients may be effective in the treatment of gynecological problems such as uterine inflammation, endometriosis, and easing symptoms of vulvovaginal atrophy in postmenopausal women by targeting inflammatory cytokines and VEGF, as previously indicated. Nonetheless, many elements of sea buckhorn activity and application remain unknown to science. Inadequate research has been conducted on the effects of sea buckhorn extract on female reproductive processes and the roles of major individual elements. There is no information concerning the possible functional interrelationship among various plant constituents in the regulation of reproductive and non-reproductive processes, for example.

The mediators of sea buckthorn action have also been studied insufficiently, whilst the role and hierarchical interrelationships between signaling molecules mediating sea buckthorn actions remain rather speculative so far. They are based mainly on similar interrelationships between mediators of other substances. For example, it is possible that plant flavonoids with antioxidant properties could block ROS, prevent oxidative stress, and resulting inflammatory processes, mutagenesis, apoptosis, and arrest cell cycle (81, 83). It is possible that this is a case of sea buckhorn isoflavones, too. Nevertheless, such mechanisms might be proposed based on indirect indications only – the action of flavones on some indices of oxidative, inflammatory processes, apoptosis, or proliferation. Furthermore, plant flavonoids usually have phytoestrogenic properties – the ability to affect the receptors of steroid hormones, which in turn are the important regulators of cell proliferation, apoptosis, and cancerogenesis (81, 84). Sea buckthorn components/molecules can affect steroid hormones and steroid hormones-dependent processes, as discussed earlier. However, it is uncertain whether steroid hormone receptors, ROS, inflammatory regulators, and so on actually cause sea buckhorn function. Although research on this plant has concentrated on its medicinal potential and use, the function of sea buckhorn extract and some of its important components on a healthy female reproductive system is still mostly unknown. It is also necessary to identify and/or standardize optimal methods of delivering biologically active molecules of sea buckhorn. This plant’s and its compounds’ medicinal potential should be validated not just *in vitro* and in animal research, but also in clinical trials. The findings of the few reported studies listed above may just be the beginning steps toward understanding the biology and therapeutic potential of this interesting plant and its active ingredients, which will require further confirmatory research.

## Author contributions

Conceptualization: MM, SR, AK. Writing – original draft preparation: MM. Writing – review and editing: SR, AK. Supervision: AK.

## Glossary

**Table:**

ALT	alanine aminotransferase
APAP	acetaminophen-induced apoptosis
AST	aspartate aminotransferase
Bcl-2/Bax	B-cell lymphoma 2/Bcl-2-associated X protein
CAT	catalase
FAS	fatty acid synthase
GSH	glutathione
GSH-Px	glutathione peroxidase
HFD	high fat diet
IDF	insoluble dietary fiber
iNOS	inducible nitric oxide synthase
IR	infrared
LDL-C	lipoprotein-cholesterol
MAPK	mitogen-activated protein kinase
MDA	malondialdehyde
MS	mass spectroscopy
NF-κB	nuclear factor kappa light chain enhancer of activated B cells
NMR	nuclear magnetic resonance
NO	nitric oxide
Nrf2	erythroid 2–related factor 2
p-Akt	phosphoinositide 3-kinase
PGC1α	peroxisome proliferator-activated receptor gamma coactivator 1
PI3K-Akt-mTOR	phosphatidylinositol 3-kinase – protein kinase B - the mammalian target of rapamycin
PP	pomace powder
PRDM 16	PR domain-containing 16
ROS	reactive oxygen species
SBSO	sea buckthorn oil
SHH	sonic hedgehog signaling pathway
SOD	superoxide dismutase
STAT	signal transducer and activator of transcription
TC	total cholesterol
TG	triglyceride
TGF β	transforming growth factor-beta
TNF α	tumor necrosis factor alpha
UCP-1	uncoupling protein 1
UV/Vis	ultraviolet/visible
VEGF	vascular endothelial growth factor
